# *A20* Overexpression Inhibits Lipopolysaccharide-Induced NF-κB Activation, TRAF6 and CD40 Expression in Rat Peritoneal Mesothelial Cells

**DOI:** 10.3390/ijms15046592

**Published:** 2014-04-17

**Authors:** Xun-Liang Zou, De-An Pei, Ju-Zhen Yan, Gang Xu, Ping Wu

**Affiliations:** 1 Department of Nephrology, the Affiliated Hospital, Hangzhou Normal University, Hangzhou 310015, Zhejiang, China; E-Mails: yanjuzhen2006@163.com (J.-Z.Y.); xugang58@aliyun.com (G.X.); apple1983819@163.com (P.W.); 2 Division of Cardiology, Hangzhou Red Cross Hospital, Hangzhou 310003, Zhejiang, China; E-Mail: smmu.peidean@126.com

**Keywords:** rat peritoneal mesothelial cells, Zinc finger protein A20, NF-κB, TRAF6, CD40

## Abstract

Zinc finger protein A20 is a key negative regulator of inflammation. However, whether A20 may affect inflammation during peritoneal dialysis (PD)-associated peritonitis is still unclear. This study was aimed to investigate the effect of A20 overexpression on lipopolysaccharide (LPS)-induced inflammatory response in rat peritoneal mesothelial cells (RPMCs). Isolated and cultured RPMCs *in vitro*. Plasmid pGEM-T easy-*A20* was transfected into RPMCs by Lipofectamine™2000. The protein expression of A20, phospho-IκBα, IκBα, TNF receptor-associated factor (TRAF) 6 and CD40 were analyzed by Western blot. The mRNA expression of *TRAF6*, *CD40*, interleukin-6 (*IL-6*) and tumor necrosis factor-α (*TNF-α*) were determined by real time-PCR. NF-κB p65 DNA binding activity, IL-6 and TNF-α levels in cells culture supernatant were determined by ELISA. Our results revealed that RPMCs overexpression of A20 lead to significant decrease of LPS-induced IκBα phosphorylation and NF-κB DNA binding activity (all *p* < 0.01). In addition, A20 also attenuated the expression of *TRAF6*, *CD40*, *IL-6* and *TNF-α* as well as levels of IL-6 and TNF-α in cells culture supernatant (all *p* < 0.05). However, A20 only partly inhibited *CD40* expression. Our study indicated that A20 overexpression may depress the inflammatory response induced by LPS in cultured RPMCs through negatively regulated the relevant function of adaptors in LPS signaling pathway.

## 1. Introduction

Continuous ambulatory peritoneal dialysis (PD) has been used as a treatment for chronic renal failure for over three decades [1]. Bacterial peritonitis is a major complication of PD and a leading cause of technique failure [2]. Clinically severe infections with gram-negative bacteria have become more frequent over the past decade, resulting in increased treatment failure and worse patient outcomes [3]. Lipopolysaccharide (LPS) derived from gram-negative organisms is a potent mediator of peritoneal inflammation and could be a trigger of the orchestrated production of chemokines.

Previous studies have identified a central role for the mesothelial cell in orchestrating peritoneal responses during inflammation and infection [4,5]. Peritoneal mesothelial cells (PMCs) have been shown to constitutively express Toll-like receptor (TLR)4. TLR4 is directly involved in LPS-induced peritoneal inflammation, in a nuclear factor-κB (NF-κB) dependent manner [6]. Recognition of LPS initiates TLR4 oligomerization, by signaling pathway, inducing IL-1 receptor-associated kinase (IRAK)4 kinase activity, IRAK4→IRAK1 phosphorylation, recruitment of TNF receptor-associated factor(TRAF)6, and engagement of TGF-β-activating kinase (TAK)1 [7–9], which activates transcription factors NF-κB, resulting in transcription of genes encoding inflammatory mediators, adhesion and costimulatory molecules [9]. The NF-κB has key functions in innate and adaptive immunity, cell survival, and development. NF-κB is activated in response to a wide range of stimuli, including proinflammatory cytokines, bacterial LPS, and viral infection [10]. In TLR4—NF-κB signaling pathway, TRAF6 is critical for LPS downstream signaling because TRAF6 knockout animals are less responsive to LPS [11].

CD40 is a member of the TNF family of receptors whose ligand (CD154) is mainly expressed on the membrane of activated CD4-positive lymphocytes. CD40 activation plays an important role in the regulation of chemokine and cytokine secretion and is a central event in major inflammatory and immune reactions [12]. Our previous studies have demonstrated that CD40 was expressed on rat peritoneal mesothelial cells (RPMCs) and may be involved in inflammatory process of the peritoneum [13,14]. And the CD40-mediated activation of NF-κB (both the canonical and noncanonical pathways) can be mediated mainly by TRAF6 [15].

Zinc finger protein A20, also known as tumor necrosis factor (TNF)-α induced protein 3 (TNFAIP3) is a cytoplasmic protein that plays a key role in the negative regulation of inflammatory response [16,17]. Experimental studies have indicated its critical role for preventing inflammation *in vivo*. A20 deficient mice spontaneously develop severe inflammation and die prematurely due to severe multi-organ inflammation and cachexia [18]. In addition, the protective role of A20 has been reported in several inflammatory diseases, including bronchial asthma, rheumatoid arthritis, and myocarditis [19–21]. However, whether A20 may affect inflammation during PD-associated peritonitis is still unclear. Therefore, this study was performed to examine the potential protective effect of A20 on preventing inflammatory response in RPMCs induced by LPS.

## 2. Results

### 2.1. Culture of RPMCs

Within three to five days following culture initiation, >95% of the cells exhibited a polygonal, cobblestone epithelioid morphology that is typical of PMCs. These cells were cytokeratin-positive and did not express factor VIII, which is consistent with a mesothelial cell phenotype (See Figure S1). These findings suggested that the proliferative cells were of a peritoneal mesothelial origin.

### 2.2. Expression of A20 Protein in RPMCs

**Figure 1.**
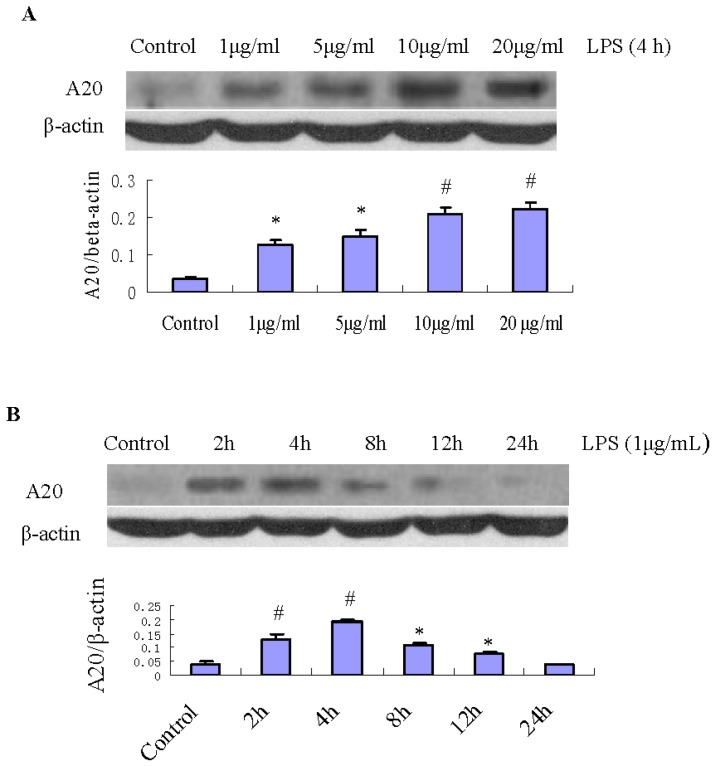

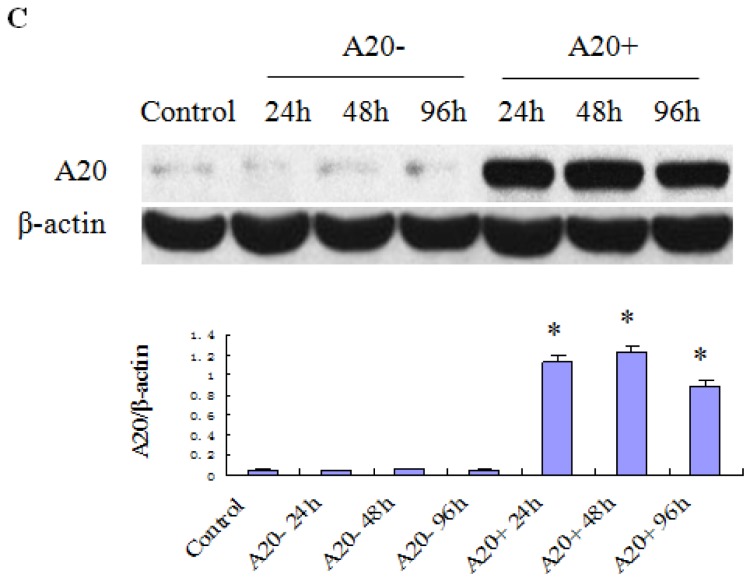
Changes in the protein expression of A20 in RPMCs after exposure to LPS. (**A**) RPMCs were treated with 1, 5, 10, and 20 μg/mL LPS for 4 h, respectively. Untreated cells were used as the control. The relative expression of A20 protein was determined by normalization to β-actin. Western blots are representative of three separate experiments. Bar graph shows the relative expression of A20. Data are presented as the mean ± SD (*n* = 3). * *p* <0.01 and ^#^ *p* < 0.005 *vs.* control; (**B**) Changes in the protein expression of A20 in RPMCs after exposure to LPS. RPMCs were treated with 1 μg/mL LPS for various times (2, 4, 8, 12, and 24 h). Untreated cells were used as the control. The relative expression of A20 protein was determined by normalization to β-actin. Western blots are representative of three independent experiments. Bar graph shows the relative protein expression of A20. Data are presented as the mean ± SD (*n* = 3). * *p* < 0.05 and ^#^ *p* < 0.01 *vs.* control; (**C**) The protein expression of A20 in RPMCs in the absence (A20−) or presence (A20+) of pGEM-T easy-*A20* (1 μg/well). RPMCs were transfected with pGEM-T easy or pGEM-T easy-*A20* for 24, 48, and 96 h, respectively. Cells incubated with serum-free DMEM/F12 alone were used as the control. The relative expression of A20 protein was determined by normalization to β-actin. Western blots are representative of three separate experiments. Bar graph shows the relative protein expression of A20. Data are presented as the mean ± SD (*n* = 3). * *p* < 0.001 *vs.* control.

A20 protein expression was increased by LPS treatment. Figure 1A shows the time course of A20 protein levels in RPMCs treated with 1 μg/mL LPS. A20 protein expression had increased at 2 h (*p* < 0.01), reached its maximum level at 4 h (*p* < 0.01), and then atenuated rapidly to the basal level at 24 h (*p* > 0.05). RPMCs treated by LPS at 1, 5, 10, and 20 μg/mL showed dose-dependent increase of A20 protein expression (Figure 1B). Densitometric analysis of Western blots demonstrated that these changes were statistically significant (*p* < 0.01). Figure 1C shows the expression of A20 protein in RPMCs in the presence or absence of the A20 overexpression plasmid (pGEM-T easy-*A20*, 1 μg/well). After transfection of pGEM-T easy-*A20* for 24 h, the expression of A20 protein had increased considerably, which was maintained to 96 h. Densitometric analysis of Western blots demonstrated that these changes were statistically significant (*p* < 0.001). Conversely, such effects were not observed in the absence of pGEM-T easy-*A20*. The long-lasting increase in A20 protein levels following the surge of the A20 transcript suggested that this protein is stable.

### 2.3. Effects of Various Treatments on RPMC Viability

**Figure 2.**
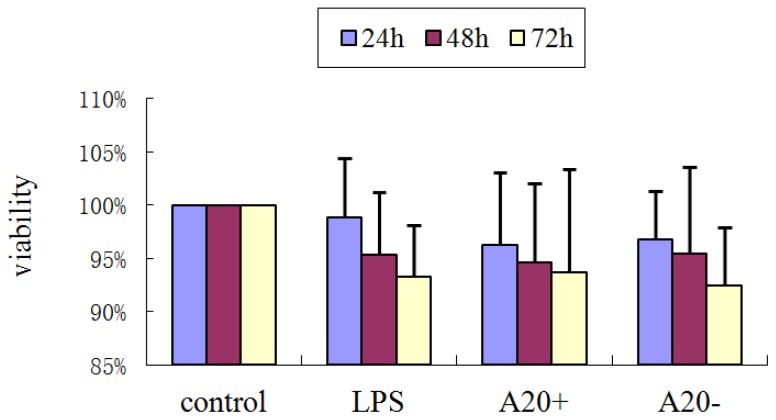
Effects of various treatments on RPMC viability. Cells were treated with serum-free DMEM/F12, serum-free DMEM/F12 + LPS, serum-free DMEM/F12 + pGEM-T easy-*A20* + LPS, and serum-free DMEM/F12 + pGEM-T easy + LPS for 24, 48, and 72 h. MTT assays were used to evaluate cell viability. Cells incubated with serum-free DMEM/F12 alone were used as the control and their cell viability was set to 100%. Data are expressed as the mean ± SD of three independent experiments. Control, LPS, A20+, and A20− represent serum-free DMEM/F12, serum-free DMEM/F12 + LPS, serum-free DMEM/F12 + pGEM-T easy-*A20* + LPS, and serum-free DMEM/F12 + pGEM-T easy + LPS, respectively. Compared with the control, there were no significant differences in cell viability (*p >* 0.05).

As shown in Figure 2, the cell viability of the control was set to 100%. The cell viabilities of LPS, A20+ and A20− were 98.82% ± 5.6%, 96.31% ± 6.8%, and 96.74% ± 4.6% at 24 h, 95.35% ± 5.9%, 94.62% ± 7.4%, and 95.44% ± 8.1% at 48 h, and 93.26% ± 4.9%, 93.71% ± 9.7%, and 92.43% ± 5.5% at 72 h, respectively. Compared with the control, we found no significant differences in cell viability (*p >* 0.05).

### 2.4. A20 Overexpression Effectively Suppresses LPS-Induced NF-κB Activation in RPMCs

**Figure 3.**
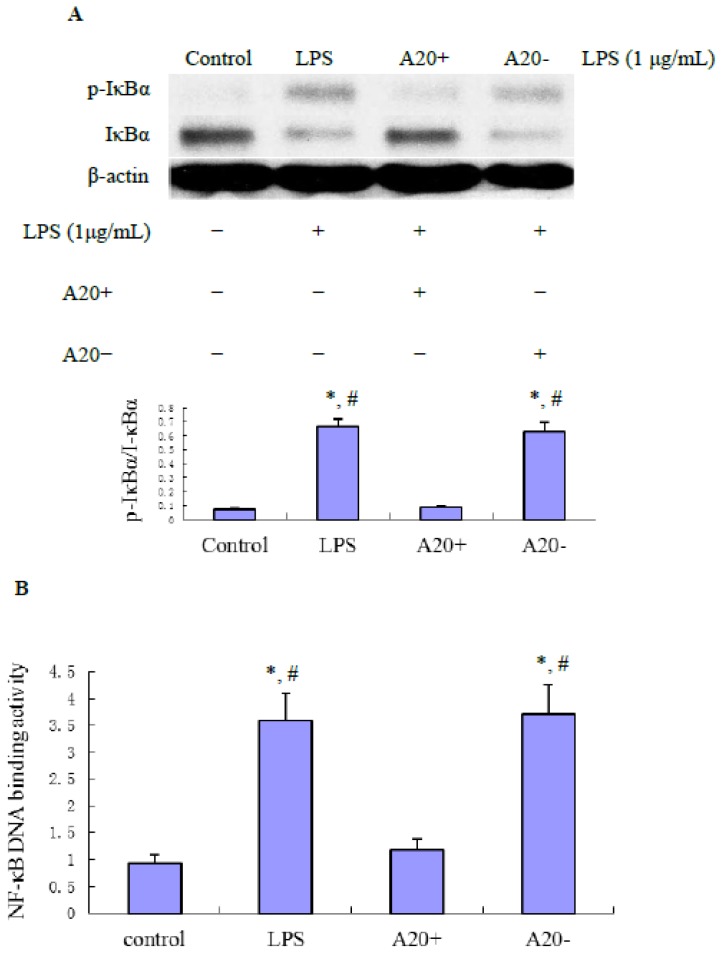
Effect of A20 on LPS-induced NF-κB signaling in RPMCs. (**A**) Cells were pre-transfected with pGEM-T easy-*A20* or pGEM-T easy for 24 h. Near confluent cells were incubated with serum-free medium for 24 h to arrest and synchronize cell growth and then exposed to LPS (1 μg/mL) for 1 h. Protein lysates were prepared and subjected to immunoblotting with antibodies against phospho-IκBα or IκBα. Western blots are representative of three independent experiments. Bar graph shows the expression of phospho-IκBα/IκBα. Data are presented as the mean ± SD (*n* = 3). * *p* < 0.01 *vs.* control; ^#^ *p* < 0.01 *vs.* A20+; (**B**) Inhibitory effect of A20 on LPS-induced NF-κB activation in RPMCs. RPMCs were pre-transfected with pGEM-T easy-*A20* or pGEM-T easy for 24 h. Near confluent cells were cultured in serum-free medium for 24 h to arrest and synchronize cell growth and then exposed to LPS (1 μg/mL) for 1 h. The DNA-binding activity of NF-κB p65 in nuclear extracts of RPMCs was measured using a NF-κB p65 transcription factor assay kit. Results were obtained from three independent experiments. Data are presented as the mean ± SD (*n* = 3). * *p* < 0.01 *vs*. control; ^#^ *p* < 0.01 *vs*. A20+.

The NF-κB pathway plays an important role in regulating the expression of proinflammatory factors during inflammatory responses. To determine whether A20 overexpression can prevent NF-κB activation, we performed immunoblot analysis of IκBα. As shown in Figure 3A, RPMCs treated with 1 μg/mL LPS for 60 min showed an increase of IκBα phosphorylation. However, IκBα phosphorylation was completely inhibited by A20 overexpression. Densitometric analysis revealed that these changes were statistically significant (*p* < 0.01). In addition, as shown in Figure 3B, the LPS-induced DNA binding activity of NF-κB was significantly decreased by transfection of pGEM-T easy-*A20* (*p* < 0.01).

### 2.5. A20 Overexpression Suppresses LPS-Induced TRAF6 Expression in RPMCs

**Figure 4.**
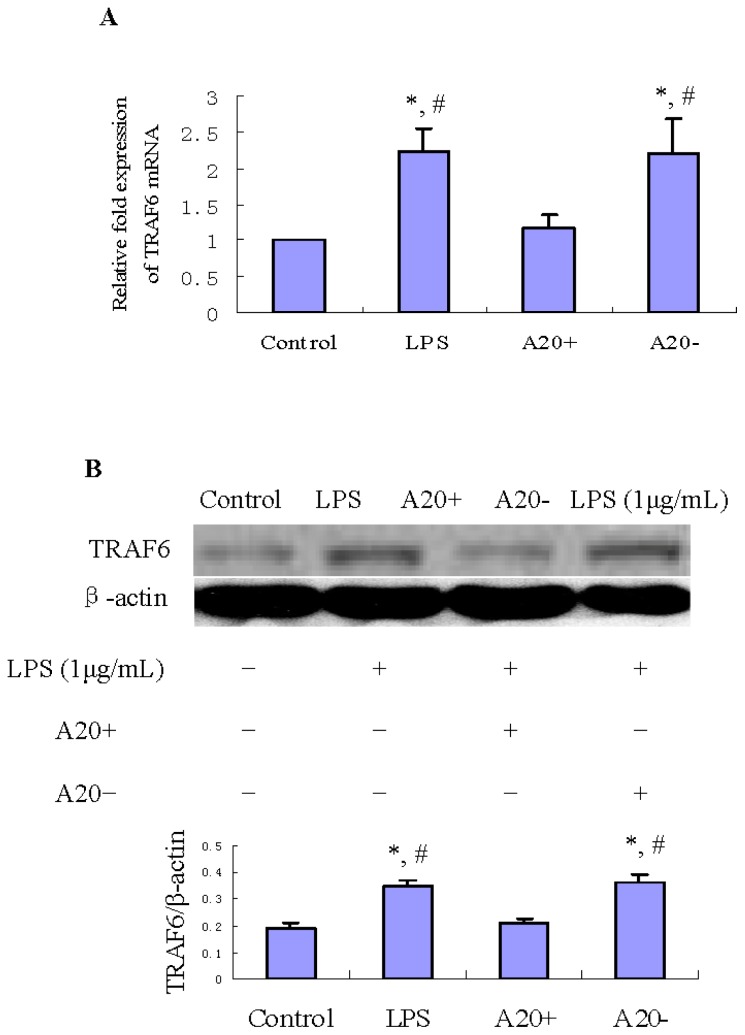
Effects of A20 on LPS-induced TRAF6 expression in RPMCs. Cells were pre-transfected with pGEM-T easy-*A20* or pGEM-T easy for 24 h. Cells incubated with serum-free DMEM/F12 alone were used as the control. Near confluent cells were incubated with serum-free medium for 24 h to arrest and synchronize cell growth and then exposed to LPS (1 μg/mL) for 1 h. RT-PCR and immunoblot analyses were performed to determine the mRNA and protein expression levels of TRAF6. The relative expression of TRAF6 protein was determined by normalization to β-actin. Results were obtained from three independent experiments. (**A**) Bar graph shows the expression of *TRAF6* mRNA. Data are presented as the mean ± SD (*n* = 3). * *p* < 0.01 *vs.* control; ^#^ *p* < 0.01 *vs.* A20+; (**B**) Bar graph shows the expression of TRAF6 protein. Western blots are representative of three independent experiments. Data are presented as the mean ± SD (*n* = 3). * *p* < 0.01 *vs.* control; ^#^ *p* < 0.01 *vs.* A20+.

TRAF6 is one of the upstream regulatory signaling molecules in the NF-κB pathway and its expression pattern is similar to that of IκBα [22]. To investigate whether A20 overexpression can suppress LPS-induced TRAF6 expression, we performed real time-PCR (RT-PCR) and immunoblot analyses of TRAF6. As shown in Figure 4A,B, treatment of RPMCs with 1 μg/mL LPS for 60 min resulted in obvious increases of *TRAF6* mRNA and protein levels compared with those in the control group (*p* < 0.01). However, A20 overexpression suppressed both the mRNA and protein levels of TRAF6 (*p* < 0.01).

### 2.6. A20 Overexpression Suppresses LPS-Induced CD40 Expression in RPMCs

**Figure 5.**
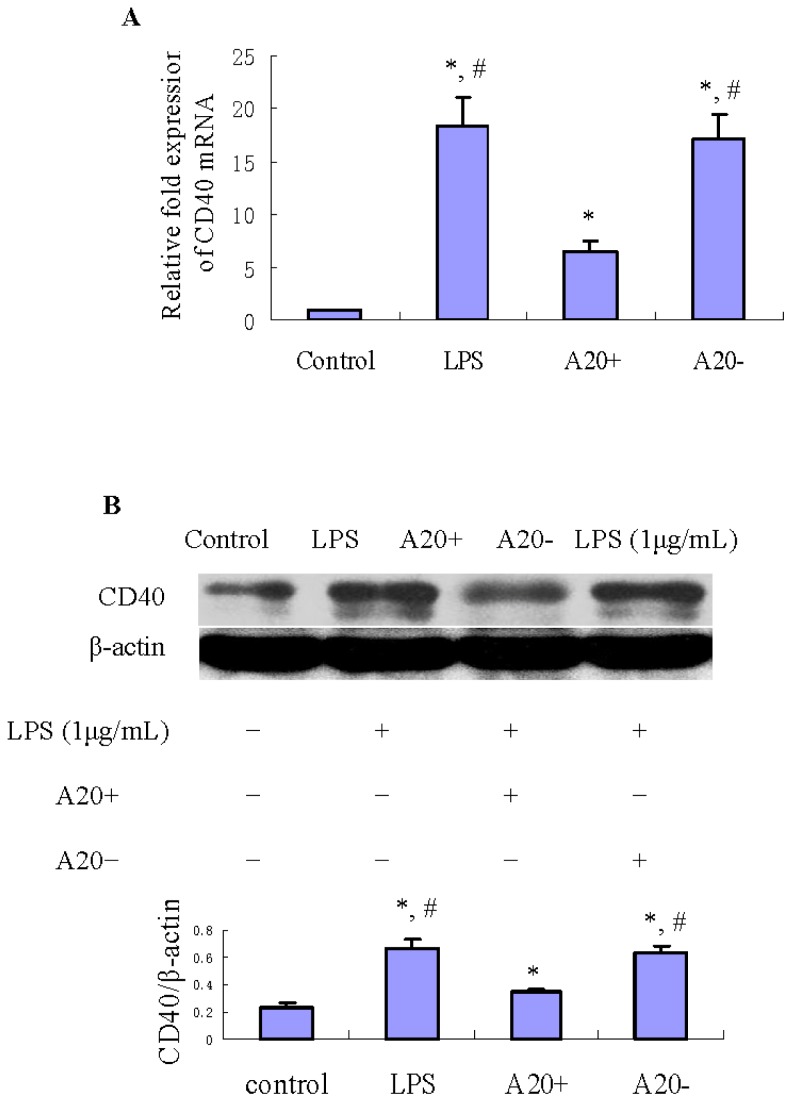
Effects of A20 on LPS-induced CD40 expression in RPMCs. Cells were pre-transfected with pGEM-T easy-*A20* or pGEM-T easy for 24 h. Cells incubated with serum-free DMEM/F12 alone were used as the control. Near confluent cells were cultured in serum-free medium for 24 h to arrest and synchronize cell growth and then exposed to LPS (1 μg/mL) for 24 h. RT-PCR and immunoblot analyses were performed to determine the mRNA and protein expression levels of CD40. The relative expression of CD40 protein was determined by normalization to β-actin. Results were obtained from three independent experiments. (**A**) Bar graph shows the expression of *CD40* mRNA. Data are presented as the mean ± SD (*n* = 3). * *p* < 0.05 *vs.* control; ^#^ *p* < 0.05 *vs.* A20+; (**B**) Bar graph shows the expression of CD40 protein. Western blots are representative of three independent experiments. Data are presented as the mean ± SD (*n* = 3). * *p* < 0.05 *vs.* control; ^#^ *p* < 0.05 *vs.* A20+.

To determine whether A20 overexpression can suppress LPS-induced CD40 expression, we performed RT-PCR and immunoblot analyses of CD40 expression in RPMCs. As shown in Figure 5A,B, treatment of RPMCs with 1 μg/mL LPS for 24 h resulted in obvious increases of *CD40* mRNA and protein levels compared with those in the control group (*p* < 0.05). However, A20 overexpression suppressed both the mRNA and protein levels of CD40 (*p* < 0.05).

### 2.7. A20 Overexpression Suppresses LPS-Induced IL-6 and TNF-α Expression in RPMCs

**Figure 6.**
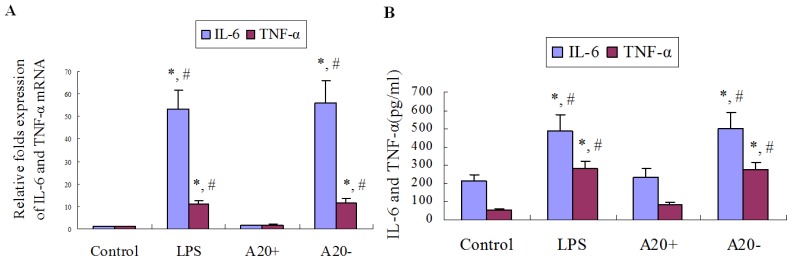
Effects of A20 on LPS-induced IL-6 and TNF-α expression in RPMCs. Cells were pre-transfected with pGEM-T easy-*A20* or pGEM-T easy for 24 h. Cells incubated with serum-free DMEM/F12 alone were used as the control. Near confluent cells were cultured in serum-free medium for 24 h to arrest and synchronize cell growth and then exposed to LPS (1 μg/mL) for 24 h. RT-PCR and ELISA were performed to determine *IL-6* and TNF-α mRNA expression as well as IL-6 and TNF-α protein levels in culture supernatants, respectively. (**A**) Bar graph shows the expression of *IL-6* and *TNF-α* mRNA. Results were obtained from three independent experiments. Data are presented as the mean ± SD (*n* = 3). * *p* < 0.01 *vs.* control; ^#^ *p* < 0.01 *vs.* A20+; (**B**) Bar graph shows the levels of IL-6 and TNF-α in culture supernatants. Results were obtained from three independent experiments. Data are presented as the mean ± SD (*n* = 3). * *p* < 0.05 *vs.* control; ^#^ *p* < 0.05 *vs.* A20+.

As shown in Figure 6A,B, treatment of RPMCs with 1 μg/mL LPS for 24 h resulted in obvious increases of *IL-6* and *TNF-α* mRNA expression levels, as well as IL-6 and TNF-α protein levels in culture supernatants compared with those in the control group (*p* < 0.05). However, A20 overexpression suppressed these effects on IL-6 and TNF-α expression (*p* < 0.05).

## 3. Discussion

In this paper, we demonstrated that RPMCs overexpression of A20 inhibited the activation of NF-κB, attenuate the expression of TRAF6 and CD40, as well as the product of IL-6 and TNF-α induced by LPS. It suggested that A20 might play a part in the local defense of the peritoneal cavity by downregulating inflammatory mediators, which may play a potential role in preventing peritoneal fibrosis induced by peritonitis.

A20 has been reported to be a rapidly inducible gene following stimulation with TLR ligands, and A20 dysfunction leads to aggravation of inflammation in various experimental models [23,24]. Here we demonstrated that LPS modulated expression of A20 in RPMCs (Figure 1). This founding suggested that A20 may contribute to the first line of defense during early stage of the inflammatory response to gram-negative bacteria.

NF-κB is critical for inflammatory responses by regulating the expression of genes such as cytokines and chemokines that drive inflammation [10]. The activity of NF-κB is tightly regulated by interaction with inhibitor of NF-κB (IκB) proteins, which are regulated by IKK-mediated IκB phosphorylation, followed by their ubiquitination and proteolysis, enabling the entry of NF-κB into the nucleus. In most cases, the activation of NF-κB is transient and cyclic upon continuous stimulation, which is due to specific negative feedback control systems, such as the NF-κB inducible synthesis of IκB and A20 proteins [25]. In human monocytes, A20 overexpression suppresses LPS-inducible IκBα degradation and activation of NF-κB within the IRAK-TRAF6-TAK1 signaling axis via interactions with IRAK1, IRAK2, and TRAF6 [26]. Our study showed that A20 overexpression suppresses LPS-inducible IκBα phosphorylation and activation of NF-κB in RPMCs. This indicated that anti-inflammation effect of A20 in PD-associated peritonitis involved inhibiting NF-κB signal pathway.

TRAF6 is an essential signaling component of TLR4 signaling [27]. Silencing of TRAF6 with siRNA caused the abrogation of the effect of LPS in RAW 264.7 macrophages. Therefore, data by Loniewski *et al.* [22] suggest that TRAF6 may have a key role in the LPS-induced inflammatory process. Jakus *et al.* [28] study showed that LPS induced transient elevation of *TRAF6* mRNA and protein level in RAW 264.7 cells, peaking at 60 min, and this increase was returned to the control level 2 h after LPS stimulation. This may involve A20 expression, as our results showed that A20 protein expression enhanced at 2 h after LPS stimulation. Furthermore, Mabilleau *et al.* [29] study also demonstrated that LPS induced the expression of A20 resulting in the degradation of TRAF6 in cultured osteoclast *in vitro*. TRAF6, an E3 ubiquitin ligase and scaffold protein, ubiquitinated TRAF6 mediates signaling that leads to the translocation of NF-κB from the cytoplasm to the nucleus, which triggers the transcription of numerous genes, including those that encode inflammatory cytokines [30]. Our study showed that in RPMCs, A20 overexpression suppresses LPS-inducible TRAF6 protein and mRNA expression.

A20 belongs to the superfamily of deubiquitinating proteases [31,32]. Recently, it has been shown that A20 forms a complex with TRAF6, leading to the deubiquitination of its Lys63-linked polyubiquitin chains and inhibition of NF-κB activation [33]. Moreover, Lin *et al.* [32] study showed that, *in vitro*, A20 might induce directly TRAF6 Lys48-linked polyubiquitination, leading to its proteasomal degradation. Mabilleau *et al.* [29] study showed that A20-TRAF6 axis has been highlighted as an essential component in preventing LPS-stimulated NF-κB activation. Levels of A20 were inversely correlated with levels of TRAF6, and Lys48-specific polyubiquitination was decreased by silencing A20. Furthermore, silencing of A20 was shown to restore TRAF6 action and NF-κB activation after LPS stimulation [33]. These results and ours indicated that block TRAF6 signaling maybe one of the functions of A20 in its anti-inflammatory effect.

Studies showed that CD40 involves immune and inflammatory responses during peritonitis [13,34]. The present results demonstrated that LPS can induce CD40 protein and mRNA expression, but these effects were attenuated by A20 overexpression in RPMCs. This suggests that *CD40* maybe a target gene for A20 *in vitro* to restrict the inflammatory response in PD-associated peritonitis. Tavares *et al.* [35] discovered that B cell specific expression of A20 restricts CD40 and B cell antigen receptor (BCR) responses, terminates CD40 triggered NF-κB signals, restricts B cell survival, and prevents autoimmunity. Like other TNF receptors (TNFR) family members, CD40 lacks intrinsic enzymatic activity and must therefore recruit cytoplasmic molecules to mediate signal transduction. Many TNFR family members interact with one or more members of a group of adapter molecules known as TRAF [36]. Ligand engagement of the receptor results in the rapid recruitment of TRAF molecules from the cytoplasm to the receptor’s cytoplasmic domain [37,38]. Bound to TNFR family proteins, at least some members of the TRAF family mediate the activation of important transcriptional regulators including NF-κB and the stress-activated protein kinases. The cytoplasmic domain of CD40 interacts with TRAF1, 2, 3, 5, and 6 [39]. Previous study indicates that TRAF6 is a positive regulator of CD40 signaling [40], therefore, A20 blocks CD40-mediated inflammatory response may involve inhibiting the expression of TRAF6 in RPMCs. However, as our results showed, A20 only partly inhibited CD40 expression, this may involve STAT-1α pathway also contributes to LPS-induced CD40 expression [41].

**Figure 7.**
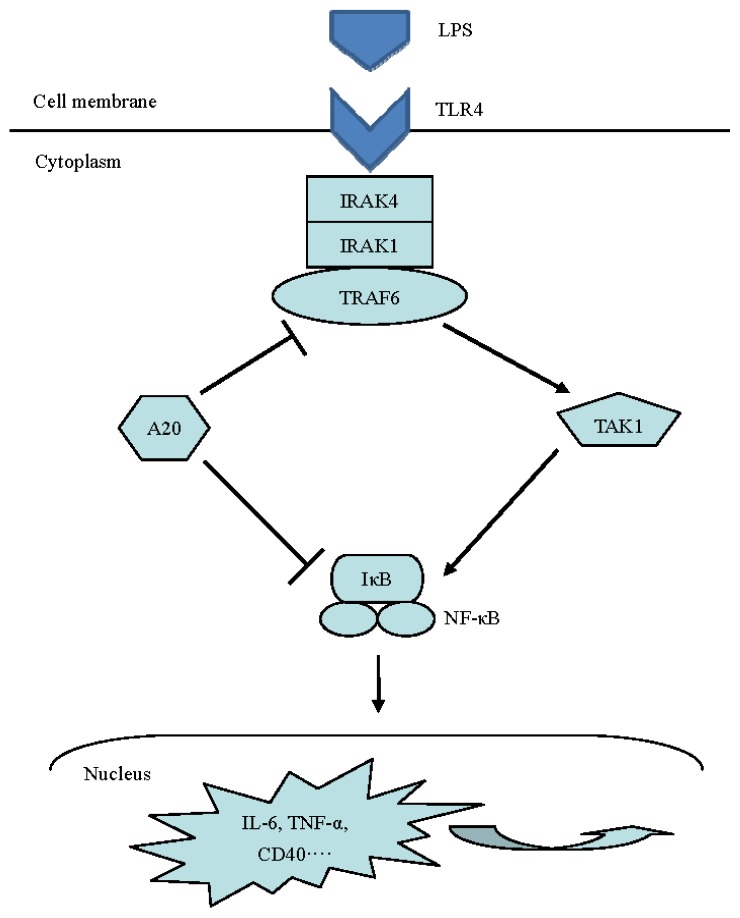
Effects of A20 on LPS signaling pathway. A20 depress the inflammatory response induced by LPS through negatively regulated the relevant function of adaptors in LPS signaling pathway.

In summary, our data suggested that A20 may has a beneficial effect on preventing LPS-induced inflammatory response in RPMCs. To our knowledge, this is the first study to demonstrate that A20 is required *in vitro* to restrict inflammatory process in PD-associated peritonitis. However, the precise molecular mechanisms by which A20 prevents LPS-induced peritonitis undoubtedly deserve further investigation. A detailed understanding of the molecular and cellular function of A20 is likely to facilitate the development of novel therapeutics for inflammatory diseases. Figure 7 summarizes the effects of A20 on LPS signaling pathway.

## 4. Materials and Methods

### 4.1. Antibodies and Reagents

Rabbit anti-rat A20 and IκBα monoclonal antibodies were purchased from Cell Signaling Technology Inc. (Danvers, MA, USA). Rabbit anti-rat CD40 polyclonal antibody was purchased from Abbiotec (San Diego, CA, USA). Rabbit anti-rat TRAF6 monoclonal antibody was purchased from Santa Cruz Biotechnology (Santa Cruz, CA, USA). LPS was purchased from Sigma (*Escherichia coli* type O111:B4; St. Louis, MO, USA). Trypsinase and the SABC reagent kit were purchased from Boster (Wuhan, China). Dulbecco’s modified Eagle’s medium (DMEM) and fetal calf serum (FCS) were purchased from Gibco-BRL (Grand Island, NY, USA). The reverse transcription kit, Lipofectamine™2000 reagent kit and TRIZOL reagent were purchased from Invitrogen (Carlsbad, CA, USA). Rat IL-6 and TNF-α ELISA kid were purchased from Bender MedSystems (Vienna, Austria) and R&D (Minneapolis, MN, USA), respectively. Plasmid pGEM-T easy-*A20* was obtained from Generay Biotechnology Co., Ltd. (Shanghai, China. The DNA sequencing of *A20* gene see Figure S2).

### 4.2. RPMCs Isolation and Culture

The isolation and culture of RPMCs was performed according to our previously reported method [13]. Briefly, Male Sprague Dawley (SD) rats weighing 150–220 g were obtained from the Experimental Animal Center of Hangzhou Normal University, Hangzhou, China. The procedure was approved by the Ethics Committee of Hangzhou Normal University. *In vitro* RPMCs were prepared by infusing 25 mL of 0.25% trypsinase-0.02% EDTA-Na_2_ into the rat abdominal cavity. The fluid was removed from the peritoneal cavity 2 h later under sterile conditions. To harvest RPMCs, cellular components were isolated by centrifugation (1500 rpm) for 10 min, then washed with D-Hank’s balanced salt solution (D-HBSS) and suspended in DMEM/F12 medium supplemented with 20% (*v*/*v*) FCS. Cells were placed into 25-cm^2^ tissue culture flasks (Corning, NY, USA) and incubated overnight at 37 °C in a humidified 5% CO_2_ atmosphere. Non-adherent cells were removed the next day with two brief washes using D-HBSS, and the adherent population was incubated at 37 °C, 5% CO_2_ in fresh culture medium. The cells reached confluence in 3–5 days. RPMCs in this study were derived from two to three passages grown as a monolayer to subconfluency.

### 4.3. Effect of LPS on Expression of A20 in RPMCs

RPMCs were passaged by dissociating the monolayer with 0.25% trypsin-0.02% EDTA-Na_2_ and were reseeded into 60-mm-diameter tissue culture plates (Corning, NY, USA). Subconfluent mesothelial cells were washed twice with fresh DMEM and incubated with FCS-free DMEM/F12 for 24 h. The cells were treated with 1 μg/mL LPS for different times (0, 2, 4, 8, 12, and 24 h) to detect A20 protein change, and different concentrations (1, 5, 10, and 20 μg/mL) for 4 h to detect A20 protein change. The expression of A20 proteins was measured by Western blot.

### 4.4. Transfection Experiments

Cells were seeded into 6-well plates at a density of 5 × 10^5^ cells/cm^2^, when cells reached 90%–95% confluency, then washed with D-HBSS and incubated with serum-free DMEM/F12. Plasmid pGEM-T easy-*A20* was transfected into cells by Lipofectamine™2000 according to the manufacturer’s instructions. An amount of 1 μg Plasmid pGEM-T easy-*A20*, and 10 μL lipofectamine, were diluted into 50 μL medium without serum respectively. Then the above two were combined and laid in room temperature for 20 min. The complexes were added onto the cells cultured with serum-free medium. Six hours later, the medium with serum was added. After transfection 24, 48, and 96 h respectively, the expression of A20 proteins was measured by Western blot.

### 4.5. Assessment of Cell Viability

RPMCs were divided into four different groups designated: control (cells incubated with serum-free DMEM/F12 alone); LPS (serum-free DMEM/F12 + LPS); A20+ (serum-free DMEM/F12 + pGEM-T easy-*A20* + LPS) and A20− (serum-free DMEM/F12 + pGEM-T easy + LPS). The plasmid pGEM-T easy-*A20* or pGEM-T easy was transfected into cells for 24 h before LPS treatment. Cells were seeded into 96-well plates at a density of 10^4^ cells/cm^2^ and cultured in DMEM supplemented with 20% FCS. Nearconfluent cells were incubated with serum-free medium for 24 h to arrest and synchronize cell growth. Afterward, LPS (1 μg/mL) was added. At selected time points (24, 48, and 72 h), MTT [3-(4,5-dimethylthiazolyl)-2,5-diphenyl-2*H*-tetrazolium bromide] was added at a final concentration of 0.5 mg/mL, and the cells were incubated for a further 4 h in a humidified environment. Dimethyl sulfoxide (DMSO) was added, and 96-well plates were gently shaken for 10 min at room temperature. The absorbance was measured by spectrophotometry at 490 nm.

### 4.6. Effect of A20 on LPS-Induced p-IκBα, IκBα, TRAF6, CD40, IL-6 and TNF-α Expression

RPMCs were reseeded into 60-mm-diameter tissue culture plates, and divided into four different groups designated as referred to above. RPMCs were transfected with plasmid pGEM-T easy-*A20* or pGEM-T easy for 24 h before LPS treatment. Nearconfluent cells were incubated with serum-free medium for 24 h to arrest and synchronize cell growth, then, exposed to LPS at 1 μg/mL for 1 and 24 h, respectively. The protein expression of p-IκBα, IκBα, TRAF6 and CD40 was measured by Western blot. The mRNA expression of *TRAF6*, *CD40*, *IL-6*, and *TNF-α* was measured by RT-PCR.

### 4.7. Western Blot Assay

Treated/untreated subconfluent RPMCs were lysed in lysis buffer (Cell Signaling Technology Inc., Danvers, MA, USA). Protein concentrations were measured using the Bradford method, and 20 μg of protein was analyzed on 10% gradient sodium dodecyl sulfate polyacrylamide gel electrophoresis (SDS-PAGE) under denaturing conditions and electro-transferred to nitrocellulose membranes. Nonspecific protein binding was blocked by incubating the membranes with blocking solution (Tris-buffered saline Tween-20 (TBST) and 5% non-fat dried milk) for 60 min at room temperature. Polyclonal antibodies specific for A20 (1:1000), IκBα (1:1000), phospho-IκBα (1:1000), TRAF6 (1:1000), and CD40 (1:1000) were applied to the membrane and incubated overnight at 4 °C. After rinsing with 1× TBST, 1:2000 diluted peroxide-conjugated anti-rabbit and anti-mouse IgG antibody was added for 60 min at room temperature. The detection of specific signals was performed using the enhanced chemiluminescence system (Cell Signaling Technology Inc., Danvers, MA, USA).

### 4.8. NF-κB DNA Binding Activity

Nuclear protein was isolated from RPMCs using the Nuclear and Cytoplasmic Extraction Reagents Kit (NE-PER). NF-κB DNA binding activity in nuclei was determined using the NF-κB p65 transcription factor assay kit according to the manufacturer’s instructions (Cayman Chemical, Ann Arbor, MI, USA). Briefly, 10 μg nuclear extracts were added to the designated wells with complete transcription factor binding assay buffer (100 μL/well) and incubated overnight at 4 °C. After washing 5 times with 200 μL wash buffer, 100 μL of 1:100 diluted NF-κB p65 antibody was added for 1 h at room temperature. After washing, 100 μL of 1:100 diluted goat anti-rabbit HRP conjugate was added to each well. Forty-five minutes later, 100 μL of transcription factor developing solution was added to each well and incubated for 15 min without light. Finally, 100 μL of stop solution was added and absorbance was measured at 450 nm.

### 4.9. Real-Time PCR

Cells were washed with HBSS and lysed in 1 mL Trizol reagent, and total RNA was prepared according to the manufacturer’s instructions. The purity and quantity of the extract were determined by UV absorption and gel electrophoresis. A total of 2 μg RNA was reverse transcribed to cDNA, using an Invitrogen first-strand synthesis kit. Quantitative RT-PCR of target cDNA was conducted for *TRAF6*, *CD40*, *IL-6*, and *TNF-α* normalized to glyceraldehyde-3-phosphate dehydrogenase (*GAPDH*) mRNA expression. Experiments were performed in 96-well plates in triplicate, using SYBR premix Ex Taq™ (Takara, Tokyo, Japan). RT-PCR amplification was performed on an ABI Prism 7000 sequence detection system (Applied biosystem, Foster, CA, USA). RT-PCR conditions were 95 °C for 30 s and then 40 cycles at 95 °C for 5 s and 55 °C for 31 s, according to the manufacturer’s instructions. Primer sequences used in this study are shown in Table 1.

### 4.10. Cytokine Assays

IL-6 and TNF-α levels in cells culture supernatants were determined by enzyme-linked immunosorbent assay (ELISA) following the manufacturers instructions.

### 4.11. Statistical Analysis

Data are expressed as mean ± SD of three independent determinations. Statistical differences among groups were assessed by one-way analysis of variance (ANOVA), followed by a Bonferroni (*post hoc*) test for continuous variables distributed normally, and by Mann-Whitney test for continuous variables without a normal distribution. A *p* value of <0.05 was considered statistically significant. The statistical calculations were performed using the statistical package SPSS for Windows 13.0 (SPSS Inc., Chicago, IL, USA).

## 5. Conclusions

In conclusion, our study indicated that A20 overexpression may depress the inflammatory response induced by LPS in cultured RPMCs through negatively regulated the relevant function of adaptors in LPS signaling pathway.

## Supplementary Information



## Figures and Tables

**Table 1.** Primer sequences for quantitative RT-PCR.

Gene	Primer sequence
*TRAF6*	Forward: 5′-GATCGGGTTGTGTGTGTCTG-3′ Reverse: 5′-AGAGACACCCCAGCAGCTAA-3′
*CD40*	Forward: 5′-GAATTCTCAGCCCAGTGGAA-3′ Reverse: 5′-GCAGGGATGACAGACGGTAT-3′
*IL-6*	Forward: 5′-AGTTGCCTTCTTGGGACTGA-3′ Reverse: 5′-CAGAATTGCCATTGCACAAC-3′
*TNF-α*	Forward: 5′-GATTATGGCTCAGGGTCCAA-3′ Reverse: 5′-CTCCCTTTGCAGAACTCAGG-3′
*GAPDH*	Forward: 5′-AACTTTGGCATTGTGGAAGG-3′ Reverse: 5′-CACATTGGGGGTAGGAACAC-3′
