# Supplementary material for: A20 Overexpression Inhibits Lipopolysaccharide-Induced NF-κB Activation, TRAF6 and CD40 Expression in Rat Peritoneal Mesothelial Cells
Source: Int J Mol Sci. 2014 Apr 17;15(4):6592–608. doi: 10.3390/ijms15046592 (PMC4013649; doi:10.3390/ijms15046592)
Supplement: Supplementary file 1 [file ijms-15-06592-s001.pdf]

Supplementary Information

Figure S1. Cells exhibited a polygonal, cobble-stone epithelioid morphology typical of peritoneal mesothelial cells (PMCs) (**A**); These cells were cytokeratin-positive and failed to express factor VIII (**B**).

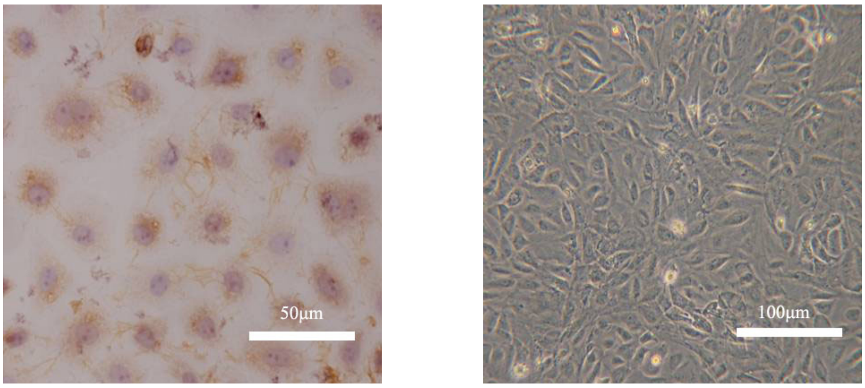

A×200 **B×400**

Figure S2. A part of DNA sequencing result of the full length *A20* gene in the recombined expression plasmid.

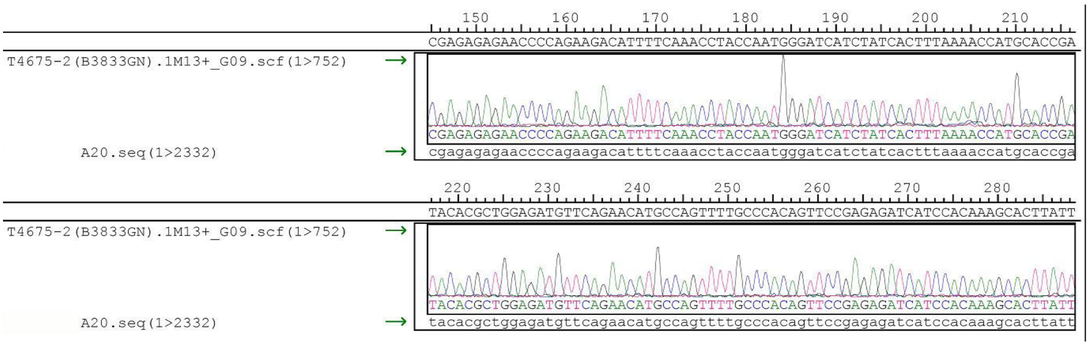

© 2014 by the authors; licensee MDPI, Basel, Switzerland. This article is an open access article distributed under the terms and conditions of the Creative Commons Attribution license (http://creativecommons.org/licenses/by/3.0/).
